# Involvement of Satellite Cell Activation via Nitric Oxide Signaling in Ectopic Orofacial Hypersensitivity

**DOI:** 10.3390/ijms21041252

**Published:** 2020-02-13

**Authors:** Jun Lee, Kinuyo Ohara, Masamichi Shinoda, Yoshinori Hayashi, Asako Kubo, Shiori Sugawara, Sayaka Asano, Kumi Soma, Kohei Kanno, Masatoshi Ando, Ryo Koyama, Yuki Kimura, Kousuke Sakanashi, Toshimitsu Iinuma, Koichi Iwata

**Affiliations:** 1 Department of Complete Denture Prosthodontics, Nihon University School of Dentistry, 1-8-13 Kandasurugadai, Chiyoda-ku, Tokyo 101-8310, Japan; iinuma.toshimitsu@nihon-u.ac.jp; 2 Department of Endodontics, Nihon University School of Dentistry, 1-8-13 Kandasurugadai, Chiyoda-ku, Tokyo 101-8310, Japan; oohara.kinuyo@nihon-u.ac.jp (K.O.); deko17008@g.nihon-u.ac.jp (K.K.); 3 Department of Physiology, Nihon University School of Dentistry, 1-8-13 Kandasurugadai, Chiyoda-ku, Tokyo 101-8310, Japan; shinoda.masamichi@nihon-u.ac.jp (M.S.); hayashi.yoshinori@nihon-u.ac.jp (Y.H.); kubo.asako@nihon-u.ac.jp (A.K.); 0629ompm@tmd.ac.jp (S.S.); desa17001@g.nihon-u.ac.jp (S.A.); sakanashi.k@gmail.com (K.S.); 4 Department of Oral Diagnosis, Nihon University School of Dentistry, 1-8-13 Kandasurugadai, Chiyoda-ku, Tokyo 101-8310, Japan; 5 Department of Pediatric Dentistry, Nihon University School of Dentistry, 1-8-13 Kandasurugadai, Chiyoda-ku, Tokyo 101-8310, Japan; deku16016@g.nihon-u.ac.jp; 6 Department of Oral Surgery, Nihon University School of Dentistry, 1-8-13 Kandasurugadai, Chiyoda-ku, Tokyo 101-8310, Japan; dema17002@g.nihon-u.ac.jp (M.A.); dery18014@g.nihon-u.ac.jp (R.K.); deyu18010@g.nihon-u.ac.jp (Y.K.)

**Keywords:** inferior alveolar nerve transection, trigeminal ganglion, mechanical allodynia, nitric oxide, satellite glial cell

## Abstract

The mechanical head-withdrawal threshold (MHWT) was significantly reduced following inferior alveolar nerve transection (IANX) in rats. Nitrate and nitrite synthesis was dramatically increased in the trigeminal ganglion (TG) at 6 h after the IANX. The relative number of neuronal nitric oxide synthase (nNOS)-immunoreactive (IR) cells was significantly higher in IANX rats compared to sham-operated and N-propyl-L-arginine (NPLA)-treated IANX rats. On day 3 after NPLA administration, the MHWT recovered considerably in IANX rats. Following L-arginine injection into the TG, the MHWT was significantly reduced within 15 min, and the mean number of TG cells encircled by glial fibrillary acidic protein (GFAP)-IR cells was substantially higher. The relative number of nNOS-IR cells encircled by GFAP-IR cells was significantly increased in IANX rats. In contrast, after NPLA injection into the TG, the relative number of GFAP-IR cells was considerably reduced in IANX rats. Fluorocitrate administration into the TG significantly reduced the number of GFAP-IR cells and prevented the MHWT reduction in IANX rats. The present findings suggest that following IANX, satellite glial cells are activated via nitric oxide (NO) signaling from TG neurons. The spreading satellite glial cell activation within the TG results in mechanical hypersensitivity of face regions not directly associated with the trigeminal nerve injury.

## 1. Introduction

It is well known that ectopic orofacial pain occurs after trigeminal nerve injuries such as tooth extraction, tooth pulpectomy, maxillofacial bone fractures, or orofacial cancers [1]. Injury-induced neuronal discharges occur after nerve damage [2], and high-frequency spikes are also generated in both uninjured and injured nerve fibers [3]. In rats, spontaneous spike discharges in uninjured nerve fibers last more than 1 week after nerve injury [3]. In an animal model of inferior alveolar nerve transection (IANX), spontaneous high-frequency activity is observed in uninjured infraorbital nerve (ION) fibers for more than 1 week after IANX, and mechanically-evoked responses in uninjured nerve fibers are also significantly higher after surgery in IANX rats compared with sham-operated animals [4]. It is likely that injured IAN fibers somehow affect the excitability of uninjured ION fibers. For a long time after the IANX, various molecules are generated in uninjured, as well as injured, trigeminal ganglion (TG) neurons, which are sensitized [5]. Neuropeptides, ATP, and chemokines are produced in TG neurons, and these molecules are released from TG neurons with injured axons [6]. Takeda et al. reported that after temporomandibular joint inflammation, substance P (SP) production is enhanced in small-diameter TG neurons, which release SP via a paracrine mechanism [7]. This SP binds to neurokinin-1 (NK1) receptors expressed by uninjured TG neurons, resulting in their enhanced excitability [7]. Various chemokines are also known to be released from injured TG neurons and are involved in the modulation of the excitability of uninjured TG neurons [8]. 

Recently, it has been reported that satellite glial cells are activated in the TG following trigeminal nerve injury [9]. Satellite glial cells tightly surround the soma of TG neurons and are involved in the modulation of neuronal excitability, as well as the nutrition of neurons [10]. Following lingual nerve crush, many cells in the TG become immunoreactive (IR) for glial fibrillary acidic protein (GFAP), a marker of activated satellite glial cells (SGCs) [11]. Many of the GFAP-IR cells in the TG also express the P2Y_12_ receptor, suggesting that ATP is released from TG neurons and binds to the P2Y_12_ receptor to activate satellite glial cells. SP, calcitonin gene-related peptide (CGRP), and tumor necrosis factor α are also released from TG neurons and involved in the activation of satellite glial cells [6]. Furthermore, connexin 43 expression is significantly enhanced in satellite glial cells suggesting that propagation of satellite glial cell activation occurs via connexin 43 [12]. Together, it is highly likely that neuron-satellite cell communication and the spread of satellite cell activation within the TG are involved in the modulation of uninjured neuronal excitability after trigeminal nerve injury. 

Nitric oxide (NO) is known to be synthesized in neurons from the amino acid L-arginine by the enzyme nitric oxide synthase (NOS). At room temperature, NO exists as a free radical gas, passes freely through biological membranes, and acts as a cell signaling factor [13]. Neuronal NOS (nNOS) is one of the three NOS isoforms that is expressed in neurons in a calcium concentration-dependent manner [14]. Sciatic nerve injury induces the upregulation of nNOS expression in dorsal root ganglion neurons that are involved in abnormal pain sensation [15]. Inhibition of nNOS diminishes injury-induced mechanical and thermal hypersensitivity associated with peripheral nerve injury [16]. In cats and dogs, many TG neurons innervating tooth pulp and gingiva express nNOS [17]. Sugiyama et al. has reported that nNOS expression is significantly accelerated in injured but not in uninjured TG neurons after IANX [18]. NO diffuses from one neuron to other neurons and affects the excitability of the target neurons [18]. In summary, following IANX, NO may be involved in neuronal communication within the TG as an atypical neuromodulator. 

Based on these findings, we hypothesized that the functional interaction between neuronal ganglion and satellite glial cells via NO-dependent mechanisms plays a pivotal role in persistent ectopic pain in the orofacial region associated with trigeminal nerve injury. To evaluate the mechanisms underlying ectopic orofacial pain after trigeminal nerve injury, we examined changes in mechanical sensitivity in the whisker pad skin of an IANX rat model. Moreover, SGC activation and nNOS expression in TG neurons and their localization within the TG were examined immunochemically. To determine whether NO-dependent SGC activation is involved in ectopic mechanical allodynia in the orofacial region following IANX, selective SGC or nNOS inhibitors were applied to the TG. Additionally, L-arginine was administrated to the TG to test whether L-arginine-induced activation of SGCs is involved in the ectopic orofacial pain. 

## 2. Results

### 2.1. Mechanical Head-Withdrawal Threshold

**Figure 1:**
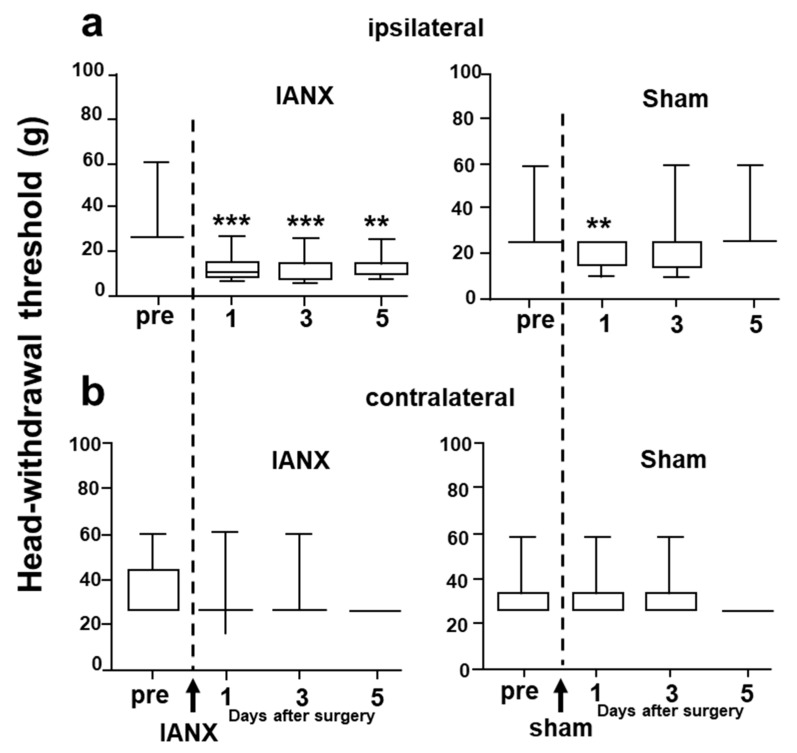
Head-withdrawal threshold to mechanical stimulation of the whisker pad skin in inferior alveolar nerve transection (IANX) and sham rats. (**a**): Ipsilateral side, (**b**): contralateral side. pre: before IANX or sham surgery, After: after IANX or sham surgery. ***: *p* < 0.001, **: *p* < 0.001 (pre vs. after). *n* = 7 in each group.

Mechanical stimulation was applied to the whisker pad skin, ipsi- or contralateral to the site of the IANX or sham surgery (Figure 1). The mechanical head-withdrawal threshold (MHWT) was significantly lower in IANX rats for 1–5 d after IANX compared to the pre-operation value ipsilateral to the IANX (Figure 1a) and ipsilateral to the sham operation was substantially lower in sham rats at 1 d after surgery. We did not observe any changes in MHWT on the side contralateral to the IANX or sham surgery. 

### 2.2. Nitrate–Nitrite Synthesis

**Figure 2:**
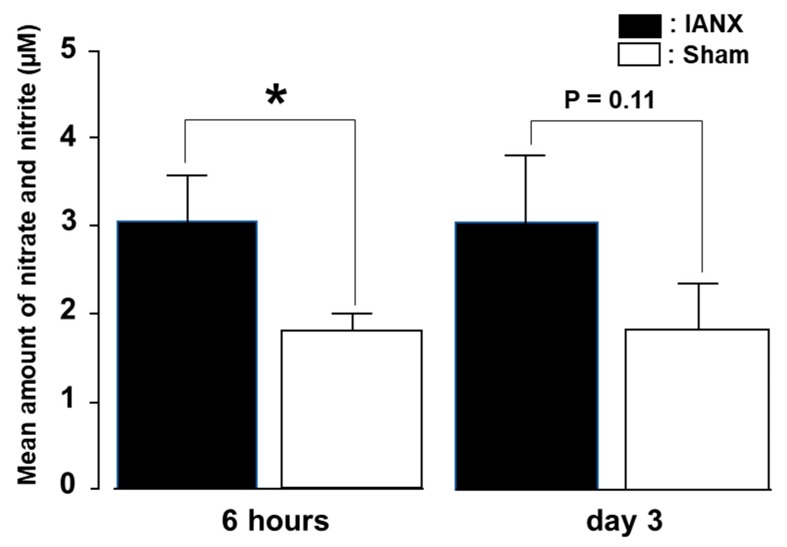
Nitrate and nitrite production in TG in sham and IANX rats. Quantitative analysis of nitrate and nitrite in TG at 6 h and on day 3 after IANX. *: *p* < 0.05. *n* = 4 in each group.

To quantify NO levels in the TG, we conducted nitrate and nitrite measurements in the TG. We observed significantly increased amounts of nitrate and nitrite in the TG of IANX rats compared to those of sham rats at 6 h after the operation (Figure 2). These levels were slightly higher in the IANX group compared to the sham group on day 3 after surgery, indicating that NO generation quickly occurs after the IANX.

### 2.3. N-Propyl-L-Arginine (NPLA) Effects on nNOS Expression and MHWT 

**Figure 3:**
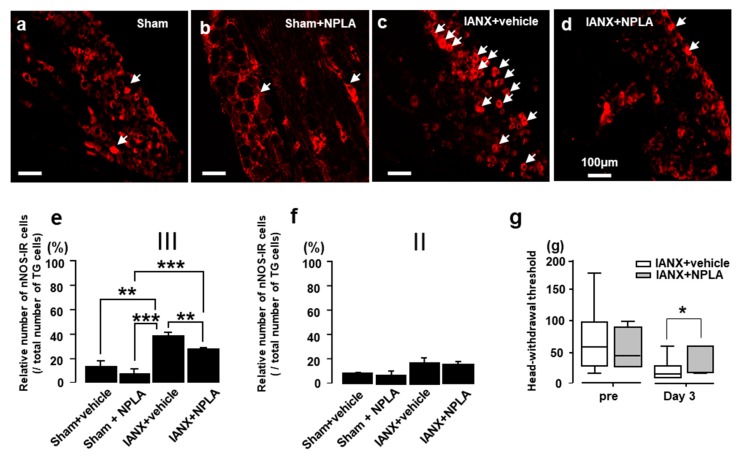
The effect of nNOS inhibitor (NPLA) administration on nNOS expression in the Ⅲ branch region of TG neurons in sham and IANX rats. (**a**): nNOS-IR cells in TG in sham, (**b**): sham+NPLA, (**c**): IANX+vehicle, (**d**): IANX+NPLA rats. (**e**): Relative number of nNOS-IR cells in the Ⅲ branch region of TG in sham, IANX+vehicle or IANX+NPLA rats. (**f**): Relative number of nNOS-IR cells in the Ⅱbranch of TG. (**g**): Effect of NPLA administration into TG on MHWT in IANX rats. The arrows indicate nNOS-IR cells. **: *p* < 0.01, ***: *p* < 0.001. *n* = 5 in each group (**e** and **f**). *: *p* < 0.05 (vehicle vs. NPLA). *n* = 7 in each group (**g**).

Next, we studied the expression of nNOS-IR in TG cells after IANX (Figure 3). We observed only low numbers of nNOS-IR cells in the TG of sham-treated rats or IANX rats exposed to NPLA. By contrast, the number of nNOS-IR cells was significantly increased in vehicle-treated IANX rats (Figure 3a–c). The relative number of nNOS-IR cells was significantly more abundant in IANX rats compared to vehicle injected sham rats and IANX rats with NPLA injection when the drugs were injected into the 3rd branch of the TG (Figure 3d), whereas no significant differences were observed when the drugs were administered to the 2nd TG branch (Figure 3e). On day 3, the MHWT of IANX rats was significantly recovered by NPLA injection into the TG (Figure 3f).

### 2.4. Effects of L-Arginine Administration on MHWT

**Figure 4:**
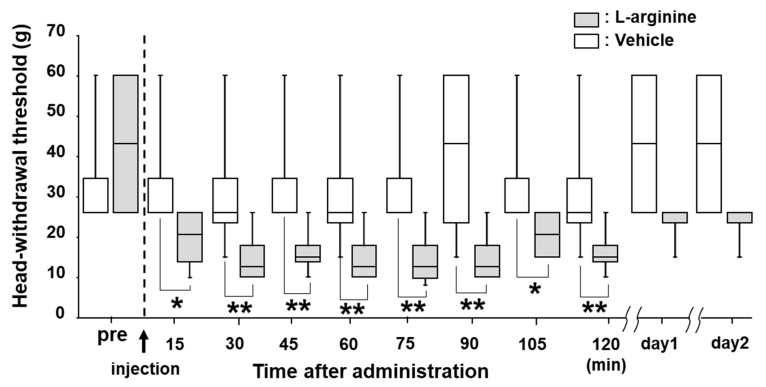
The effect of L-arginine administration into TG on MHWT in naïve rats. Time-course change in HWT to mechanical stimulation of the whisker pad skin following L-arginine administration. *: *p* < 0.05, **: *p* < 0.01. *n* = 5 in each group.

L-arginine is a substrate for the enzyme nNOS that synthetizes NO [13]. Thus, we injected L-arginine into the TG of naïve rats to examine whether mechanical allodynia was induced by L-arginine administration. After L-arginine injection into the TG, the MHWT was significantly reduced after 15 min. This decrease in MHWT lasted 120 min, but the MHWT changes were fully reversed to pre-injection levels on days 1 and 2 after the drug administration (Figure 4).

### 2.5. TG Cells Encircled by GFAP-IR Cells after L-Arginine Administration

**Figure 5:**
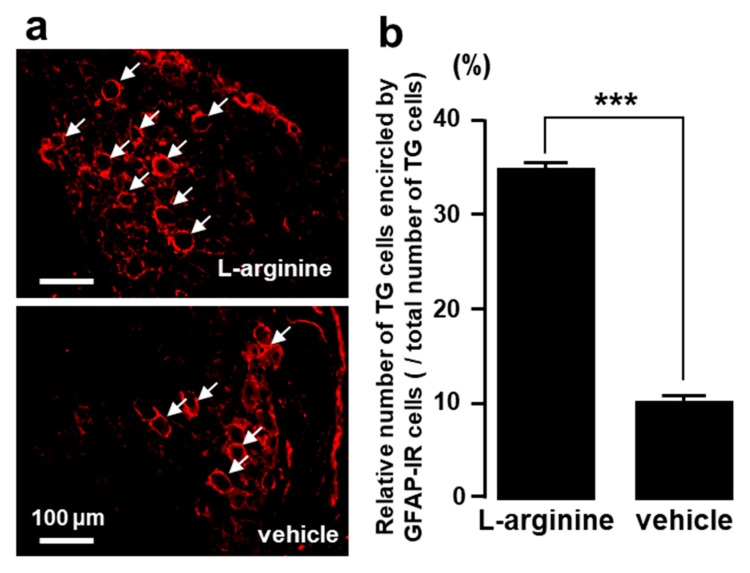
The effect of L-arginine administration into TG on the expression of GFAP-IR cells in TG. (**a**): Photomicrographs of GFAP-IR cells in TG, (**b**): Relative number of TG cells encircled by GFAP-IR cells. The arrows indicate TG cells encircled by GFAP-IR cells. ***: *p* < 0.001. *n* = 5 in each group.

We observed many GFAP-IR cells encircling TG cells 6 h after L-arginine but not vehicle administration into the TG (Figure 5a). The mean number of TG cells surrounded by GFAP-IR cells was significantly higher in L-arginine-treated rats compared to vehicle-administrated rats (Figure 5b).

### 2.6. nNOS-IR Cells Encircled by GFAP-IR Cells

**Figure 6:**
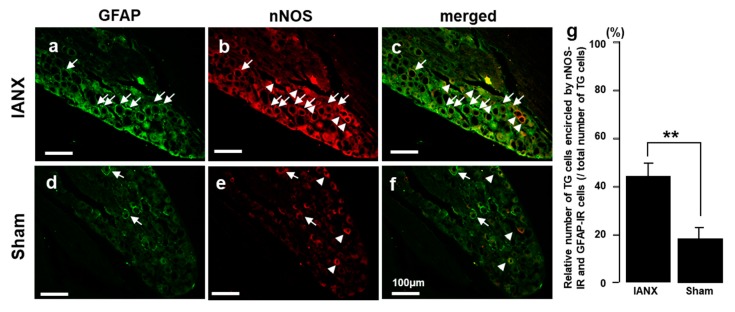
nNOS expression in GFAP-IR cells after IANX or sham surgery. (**a**–**f**): Photomicrographs of GFAP-IR cells and nNOS-IR cells in III branch region of TG in IANX and sham rats. (**g**): Relative number of GFAP-IR cells and nNOS-IR cells in III branch region of TG in IANX and sham rats. The arrows indicate GFAP-IR+ nNOS-IR cells, and arrowheads indicate nNOS-IR cells. ** *p* < 0.01. *n* = 5 in each group.

We also examined nNOS-IR cells surrounded by GFAP-IR cells in the TG of IANX and sham-operated rats. Many nNOS-IR cells were encircled by GFAP-IR cells in the TG of IANX rats, but only a few of these cells were observed in sham-operated rats (Figure 6a–f). The relative number of nNOS-IR cells encircled by GFAP-IR cells in the 3rd branch region of the TG was significantly increased in IANX rats compared to sham rats (Figure 6g).

### 2.7. Effects of NPLA on GFAP Expression in the TG

**Figure 7:**
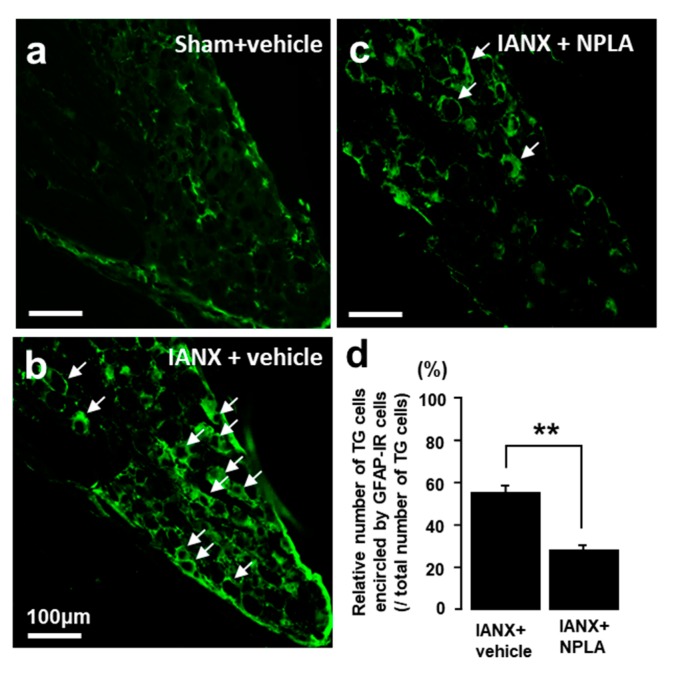
The effect of nNOS inhibitor (NPLA) injection on GFAP expression in TG in sham and IANX rats on day 3. (**a**): GFAP-IR cells in sham+vehicle (**b**) or IANX+NPLA rats (**c**). (**d**): Relative number of TG cells encircled by GFAP-IR cells in INAX+vehicle, IANX+NPLA and sham+vehicle rats. The arrows indicate GFAP-IR cells. ** *p* < 0.01, *n* = 5 in each group.

In sham-operated rats, only a small number of GFAP-IR cells were observed in the TG (Figure 7a). By contrast, many GFAP-IR cells were detected in the TG after IANX (Figure 7b). After NPLA injection into the TG, the relative number of GFAP-IR cells was significantly reduced in IANX rats in comparison with vehicle-treated IANX rats (Figure 7c,d). 

### 2.8. Effects of Fluorocitrate (FC) on GFAP Expression and MHWT

**Figure 8:**
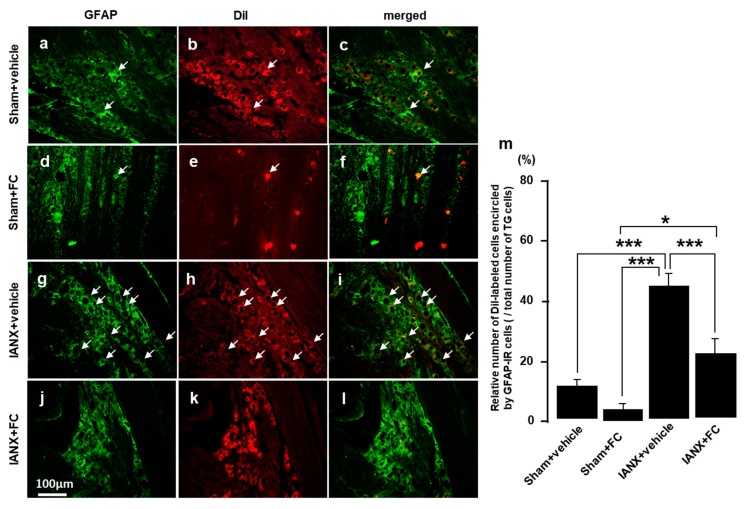
The effect of FC injection into TG on GFAP expression in TG in sham and IANX rats. (**a**–**c**): sham+vehicle, (**d**–**f**): sham+FC, (**g**–**i**): IANX+vehicle, (**j**–**l**): IANX+FC rats. (**m**): Relative number of GFAP-IR cells in sham+vehicle, sham+FC, IANX+vehicle or IANX+FC. FC: aconitase inhibitor. The arrows indicate DiI-labeled cells encircled by GFAP-IR cells. * *p* < 0.05, *** *p* < 0.001. *n* = 5 in each group.

Finally, we studied the expression of GFAP in the TG of IANX rats in the presence and absence of FC. A small number of GFAP-IR cells were observed in the TG of sham-operated rats, and no TG cells were encircled by GFAP-IR cells (Figure 8a–c). 

**Figure 9:**
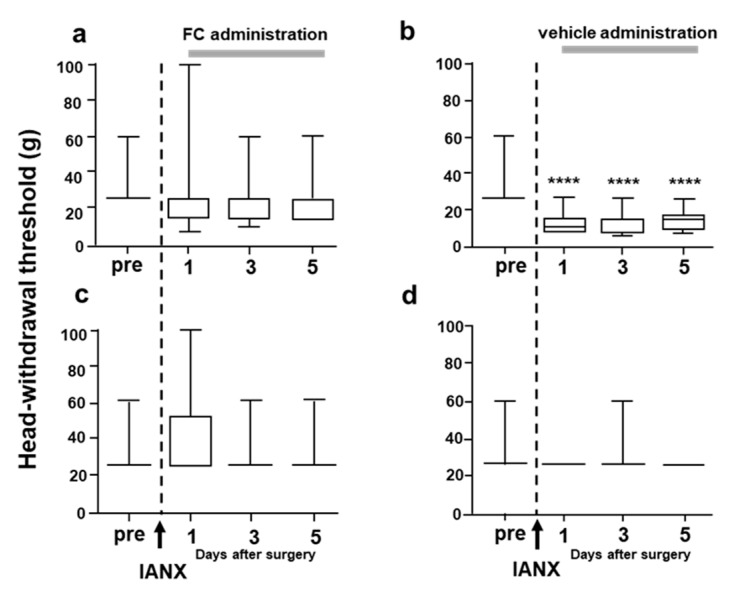
The effect of FC administration into TG on MHWT in IANX rats. (**a**,**b**): Ipsolateral side, (**c**,**d**): contralateral side. (**a**,**c**): IAN+FC, (**b**,**d**): IANX+vehicle. pre: before IANX. **** *p* < 0.0001 (pre vs. after). *n* = 7 in each group. Grey lines indicate the period treated FC and vehicle.

In contrast, many GFAP-IR cells were observed in vehicle-treated IANX rats, and many of them encircled 1,1′-Dioctadecyl-3,3,3′,3′-tetramethylindocarbocyanine perchlorate (DiI)-labeled TG cells (Figure 8d–f). The relative number of DiI-labeled cells encircled by GFAP-IR cells was significantly higher in vehicle-treated IANX rats compared to sham-operated (+vehicle or +FC) animals and FC-treated IANX rats (Figure 8j). The MHWT was also reversed to pre-IANX levels after FC administration into the TG (Figure 9a), whereas the MHWT was significantly reduced in IANX rats following vehicle administration into the TG (Figure 9b). We could not observe any changes in MHWT on the side contralateral to the IANX (Figure 9c,d). 

## 3. Discussion

The findings of the present study are summarized as follows. (1) The head-withdrawal threshold to mechanical stimulation of the whisker pad skin was significantly reduced following IANX. (2) Nitrate and nitrite levels were significantly increased in the TG after IANX, and nNOS was also considerably expressed in these neurons. (3) Inhibition of nNOS expression caused suppression of glial cell activation and reduction in MHWT in IANX rats. (4) Following L-arginine injection into the TG, the MHWT was considerably reduced, and the mean number of TG cells encircled by GFAP-IR cells was significantly higher in L-arginine-treated rats. (5) The number of nNOS-IR cells encircled by GFAP-IR cells was significantly increased following IANX. (6) The blockade of satellite glial cell activation caused significant recovery of the MHWT in IANX rats.

These observations suggest that NO is a key molecule involved in neuron-satellite glial cell communication and associated with mechanical allodynia in the broad orofacial area following trigeminal nerve injury.

### 3.1. Satellite Glial Cell Activation

Trigeminal nerve injury is known to cause mechanical allodynia and thermal hyperalgesia in the orofacial region, namely orofacial neuropathic pain [19]. To evaluate the mechanisms underlying this pain, a variety of animal models of trigeminal nerve injury have been developed [20]. The present experiment was conducted using a rat model with IANX. This IANX model mimics the inferior alveolar nerve injury following tooth extractions or other surgical treatments of the lower jaw in humans [21]. In this model, the mechanical hypersensitivity spreads over a wide area in the face extending to the uninjured 1st and 2nd branch regions of the trigeminal nerve [4]. 

We observed a significant reduction in the MHWT following IANX, as reported in a previous study [22], indicating that hypersensitivity to mechanical stimulation of the facial skin extends to uninjured areas. In the lingual nerve crush model, Katagiri et al. reported that the satellite glial cell activation spreads over a broad area within the TG [11]. We made a similar observation that after IANX, many GFAP-IR cells were detected in broad areas of the TG [12]. Furthermore, FC administration caused a significant recovery of the lowered MHWT in IANX rats. Taken together, these findings suggest that widespread satellite glial cell activation in the TG is involved in the spreading of TG neuron hyperexcitability, resulting in mechanical hypersensitivity of large areas of the face [12]. 

### 3.2. NO Synthesis

NO is thought to act as a neurotransmitter in peripheral and central nervous systems and is synthesized from L-arginine and oxygen by the enzyme NOS in ganglion neurons following nerve injury or tissue inflammation [23]. NO is quickly released from neurons and permeates the neuronal membrane, resulting in the modulation of nociceptive neuronal activity [18]. Administration of the nitric oxide synthase inhibitor N omega-Nitro-L-arginine methyl ester hydrochloride (L-NAME) causes the inhibition of neuropathic pain in rats, indicating that NO is involved in the induction of neuropathic pain associated with peripheral nerve injury [24]. 

NO is also known to enhance the release of SP and CGRP from C-fiber terminals, thus contributing to secondary hyperalgesia in tissues [25]. Furthermore, a low dose injection (0.1–1 μg) of L-arginine into the hind paw intensifies the second phase of formalin-induced hyperalgesia [26]. In agreement with these findings, we observed that nitrate and nitrite, which are metabolic products synthesized from NO, were significantly more abundant in IANX rats compared to sham rats. Moreover, many TG neurons expressed nNOS in our IANX model rats, and L-arginine administration into the TG caused a significant increase in the number of nNOS-positive neurons in the TG. Together with previous data, the present results suggest that NO synthesized in TG neurons of IANX rats is involved in persistent orofacial pain associated with orofacial inflammation and trigeminal nerve injury. 

### 3.3. NO and Satellite Glial Cell Activation

After IANX, high-frequency discharges are generated in uninjured, as well as injured, neurons [4]. It is accepted that these injury-induced discharges last for several days after a peripheral nerve injury. Various molecules synthesized by ganglion neurons are involved in this phenomenon [5] with NO being considered one of these molecules [18]. NO is also known to have a unique function for nociceptive transmission in the primary afferents and spinal dorsal horn [27]. In the central nervous system, NO synthesis also occurs following activation of neurons. NO is released from neurons and permeates into glial cells, resulting in microglial and astroglial activation [28]. This mechanism is thought to occur in primary afferent neurons, in which activation of ganglion neurons enhances NO synthesis. This synthesized NO is released from somata, as well as the peripheral and central terminals of ganglion neurons, resulting in the activation of satellite glial cells. 

In our study, nNOS positivity was detected in injured but not uninjured TG neurons of IANX rats. Furthermore, injection of the NO synthesis inhibitor NPLA into the TG caused a significant decrease in the number of nNOS-IR and GFAP-IR cells in the TG. Similarly, IANX-induced MHWT changes were significantly reversed following NPLA administration. This series of events underlying NO-mediated neuron-satellite glial cell communication causing the spreading of satellite glial cell activation into uninjured TG branch regions may be involved in the observed hyperexcitability of uninjured TG neurons following trigeminal nerve injury. 

In cell culture studies, CGRP secreted from neurons has also been reported to induce an increase in inducible NOS (iNOS) in SGCs via mitogen-activated protein kinase signaling pathways [29,30]. NO released from SGCs can activate soluble guanylate cyclase to increase the intracellular cyclic guanosine monophosphate (cGMP) concentration in ganglion neurons. Subsequently, the cGMP-dependent protein kinase G activates and modulates numerous types of target molecules by phosphorylation [31], resulting in the activation of SGCs encircling both injured and uninjured TG neurons. 

## 4. Materials and Methods

### 4.1. Animals

Male Sprague Dawley rats (SCL, Inc., Shizuoka, Japan) (*n* = 112, 7 weeks old, weighing 200–250 g) were used in this study. The animals were housed in cages (42 × 26 × 22 cm) that were kept at constant room temperature (23 ± 2 °C) and relative humidly (55 ± 5%) under conditions of a 12-h light/dark cycle (lights on at 7:00 a.m.) with free access to food and drinking water. The care and treatment of the animals used in this study were conducted according to the guidelines of the International Association for the Study of Pain [32], and all procedures were approved by the Animal Experimentation Committee at Nihon University (Protocol No. AP16D003 and AP16D044, both are accepted on 28 February 2018).

### 4.2. Inferior Alveolar Nerve Transection

For the surgical procedure of the IANX, the rats were deeply anesthetized by intraperitoneal (i.p.) administration of a saline solution containing 2.5 mg/kg butorphanol (Meiji Seika Pharma, Tokyo, Japan), 0.375 mg/kg medetomidine (Zenoaq, Fukushima, Japan), and 2.0 mg/kg midazolam (Sandoz, Tokyo, Japan). The animals were placed on a warm mat (37 °C). The left facial skin and masseter muscle were incised, and the surface of the alveolar bone was exposed. The alveolar bone covering the inferior alveolar nerve was removed to expose the IANX, and was tightly ligated with a 4-0 silk thread at two points and transected between the two ligatures and repositioned in the mandible canal without a discernable gap between the cut nerve ends. For control, a sham operation was identical to that described above but without the transection. The incised skin was sutured using a 6-0 silk thread.

### 4.3. Behavioral Assessment

Prior to behavioral testing, the rats were trained to keep their head protruding from a hole in a plastic cage while the left whisker pad skin was mechanically stimulated with von Frey filaments (North Coast Medical Inc., Morgan Hill, CA, USA). To quantitatively evaluate the MHWT, we applied 4, 6, 8, 10, 15, 26, and 60 g of mechanical stimuli to the whisker pad skin. The MHWT was determined as the minimum stimulation intensity that evoked a head-withdrawal behavior in response to more than 3 out of 5 stimuli. The MHWT was measured for 5 d after IANX or sham operation. All behavioral tests were conducted under blinded conditions 

### 4.4. Measurement of Nitrate and Nitrite

NO is known to exist in vivo as an unstable free radical gas that passes freely through biological membranes and acts as a cell signaling factor. Therefore, we measured serum NO metabolites (nitrate/nitrite) as a quantification of the NO level [33]. The rats were transcardially perfused with 100 mL saline at 4 °C under deep anesthesia at 6 h and on day 3 after IANX or sham operation. The TG was removed, homogenized, and centrifuged at 4 °C in 0.01 M phosphate-buffered saline (PBS). Total nitrate and nitrite in the extracted supernatants from homogenized TGs were measured using a nitrate/nitrite colorimetric assay kit (Cayman Chemical, Ann Arbor, MI, USA) as previously described [18]. Absorbance values were obtained by subtracting the background value to correct for absorbance attributable to non-specific binding, and was measured using an iMark microplate absorbance reader (Bio-Rad, Hercules, CA, USA).

### 4.5. Drug Administration into the TG

Rats were anesthetized with sodium pentobarbital (50 mg/kg, i.p.) and placed in a stereotaxic frame, the skull was exposed, and a small hole (diameter: 1 mm) was drilled directly above the location of the bifurcation between the 1st and 2nd branches of the TG (V1/V2) and the 3rd branch of the trigeminal nerve region (V3) of the TG. Through this hole, a guide cannula was implanted in the TG ipsilateral to the IANX or sham operation and fixed to the skull with stainless screws and dental acrylic cement. The coordinates for the target site of the TG were 9 mm below the skull surface, 2.8 mm anterior from the posterior fontanelle, and 2.7 mm lateral to the surgical suture. The rats were allowed to recover from the operation for a week. 

Fluorocitrate (FC; 100 fmol; Sigma-Aldrich, St. Louis, MO, USA), an aconitase inhibitor that is taken up by glial cells and leads to inhibition of the glial tricarboxylic acid cycle, was dissolved in PBS (vehicle). For continuous FC or vehicle administration into the TG, rats were anesthetized, and a 31-gauge injection needle was inserted through the guide cannula 9.5 mm below the skull and was connected to an osmotic pump (ALZET Pump Model, Durect, Cupertino, CA, USA) filled with FC or vehicle by a soft micro-silicone tube. The osmotic pump was embedded in the dorsal body part of the rat. FC (1 µL/h) was administered for 3 d continuously from the osmotic pump.

For vehicle (10 µl, saline) or N-propyl-L-arginine hydrochloride (NPLA; 5 µL, 0.4 µM; Tocris Bioscience, Bristol, UK) administration into the TG, a 31-gauge injection needle was inserted in the TG 9.5 mm below the skull surface through the guide cannula under light anesthesia with 2% isoflurane in oxygen. Then, 5 µL of NPLA or vehicle was administered into the TG once per day for 3 d after IANX or sham operation. Saline as a vehicle or L-arginine (10 µL, 0.5 µM; Sigma-Aldrich) dissolved in saline was also administrated into the TG in naïve rats.

### 4.6. Immunohistochemistry

To identify the TG neurons innervating the left whisker pad skin, 100 mg/mL 1,1′-dioctadecyl-3,3,3′,3-tetramethylindocarbocyanine methane sulfonate (DiI; Molecular Probes, Eugene, OR, USA; dissolved in 100% ethanol) was used as a retrograde labeling tracer. DiI was injected into the skin of the left whisker pad with a 30-gauge needle 7 d before and 3 d after IANX or sham operation, rats with continuous administration of FC or vehicle, or rats with once per day for 3 d administration of NPLA or vehicle into the TG, were anesthetized with sodium pentobarbital (50 mg/kg. i.p.) and transcardially perfused with 100 mL saline followed by a fixative containing 4% paraformaldehyde in 0.1 M PBS at 4 °C (pH 7.4). The TGs were removed after perfusion, immersed in the same fixative, and transferred to 20% sucrose in 0.01 M PBS for 12 h at 4 °C for cryoprotection. The TGs were embedded in Tissue Tek (Sakura Finetechnical, Tokyo, Japan) and stored at −20 °C for cryosection, and were cut on the horizontal plane along the long axis at a thickness of 10 µm. Every 10th section was selected, thaw-mounted on MAS-GP micro slide glass (Matsunami, Osaka, Japan), and then dried at room temperature. After rinsing with 0.01 M PBS, the sections were incubated with mouse anti-GFAP monoclonal antibody (1:500; Merck Millipore, Billerica, MA, USA) in 0.01 M PBS containing 4% normal goat serum and 0.3% Triton X-100 (Sigma-Aldrich) for 3 d at 4 °C or with goat anti-nNOS polyclonal antibody (1:500, Abcam, Cambridge, MA, USA) in 0.01 M PBS containing 4% normal donkey serum and 0.3% Triton X-100 overnight at 4 °C. After rinsing with 0.01 M PBS, the sections were reacted with AlexaFluor 488-conjugated goat anti-mouse IgG (1:200 in 0.01 M PBS) or AlexaFluor 594-conjugated donkey anti-goat IgG (1:200 in 0.01 M PBS) for 2 h at room temperature, respectively. After another rinse with 0.01 M PBS, sections were coverslipped with mounting medium (Thermo Fisher Scientific, San Jose, CA, USA) and examined using a fluorescence microscope BZ-9000 system (Keyence, Osaka, Japan). The number of DiI-labeled TG neurons encircled by GFAP-immunoreactive cells over 1/2 of the TG cell body was counted as GFAP-IR, and the number of nNOS-immunoreactive TG neurons expressing twice or greater the luminescence intensity relative to the background was counted as nNOS-IR. We also studied double immunohistochemistry with nNOS-IR and GFAP-IR cells in the TG. nNOS-IR cells encircled by GFAP-IR cells were counted in the TG of sham-operated and IANX rats, and the relative number of nNOS-IR cells encircled by GFAP-IR cells was calculated (total number of nNOS-IR cells encircled by GFAP-IR cells/total number of nNOS-IR cells × 100). No specific labeling was observed in the absence of primary antibodies.

### 4.7. Statistical Analysis

Behavioral data are presented as box and whisker plots. The box bottom and top indicate the lower and upper quartiles, respectively, and the lower and upper whiskers represent the minimum and maximum values, respectively. Other results are expressed as the mean ± SEM, and statistical analyses were performed using Student’s *t*-test for the quantitative analysis of nitrate and nitrite. The one-way repeated-measures analysis of variance (ANOVA) was followed by Bonferroni’s, Tukey’s, or Dunnet’s multiple comparison tests, as appropriate. Data were considered significant at *p* < 0.05.

## 5. Conclusions

The present findings suggest that IANX leads to satellite glial cell activation via NO, which is released from TG neurons. The activation of satellite glial cells spreads to large areas within the TG and can affect uninjured branches of the trigeminal nerve, resulting in mechanical hypersensitivity of orofacial regions not directly associated with the trigeminal nerve injury. 

The present findings suggest that NO signaling in the trigeminal ganglion is a therapeutic target for the treatment of orofacial neuropathic pain.
